# Growth of Human Hair

**Published:** 1899-10

**Authors:** 


GROWTH OF HUMAN HAIR.—Authorities differ as to the rate of growth of the human hair, and it is said to be very dissimilar in different individuals. The most usually accepted calculation gives six and one half inches per annum. A man's hair, allowed to grow to its extreme length, rarely exceeds twelve or fourteen inches, while that of a woman will grow in rare instances to seventy inches or seventy-five inches, though the average does not exceed twenty-five to thirty inches.

PULP CAPPING.—A pulp cap must be a disinfectant; an antiseptic; an antiphlogistic, and a non-conductor of thermal changes. To secure the combination apply rubber-dam; remove all debris; saturate cavity with creosote and wipe dry; introduce iodoform, followed by copal-ether varnish a little thicker than cream. Cut asbestos paper to cover; press gently down and varnish over. Oxyphosphate of zinc over this.

THE ANTHRAX BACILLUS DOES NOT FORM TOXINS.—"We have no evidence to prove the general assumption that the anthrax bacillus generates a toxin. On the contrary, everything tends to indicate that the anthrax bacillus is a typical infectious micro-organism."—*Journal American Medical Association.*

IT is remarkable how much salt water the colon will absorb, and that quickly, almost greedily. In a primipara in collapse after labor one pint of saline solution was injected hourly for twenty-four hours, all being absorbed, and the patient quickly rallied.—*N. Y. Med. Times.*

